# High-Protein Concentrated Pro-Yogurt (Pro-WPI) Enriched With Whey Protein Isolate Improved Athletic Anemia and Performance in a Placebo-Controlled Study

**DOI:** 10.3389/fnut.2021.788446

**Published:** 2022-01-20

**Authors:** Mohamed A. E. Gomaa, Marwa G. Allam, Abdallah A. I. M. Haridi, Alaa-Eldin M. Eliwa, Amira M. G. Darwish

**Affiliations:** ^1^Department of Food Science, Faculty of Agriculture, Saba Basha, Alexandria University, Alexandria, Egypt; ^2^Department of Sport Medicine, Russian State University for Physical Education, Sport, Youth and Tourism (SCOLIPE), Moscow, Russia; ^3^Department of Biological Sciences and Sports Health, Faculty of Physical Education for Men, Alexandria University, Alexandria, Egypt; ^4^Department of Food Technology, Arid Lands Cultivation Research Institute, City of Scientific Research and Technological Applications (SRTA-City), Universities and Research Center District, Alexandria, Egypt

**Keywords:** high-protein pro-yoghurt, athletes' anemia, athletic performance, urinary albumin, randomized placebo controlled

## Abstract

Upcoming developments are attracting attention to both high-protein and probiotics supplementation for the sports community to promote good health and exercise performance. This study aimed at the production of high-protein concentrated pro-yogurt (Pro-WPI) enriched with 10 and 20% whey protein isolate (WPI) and investigation of the response of daily consumption on anthropometric, hematology parameters, and athletic performance in parallel with safety consideration assessment. Twenty-four athletes (19.6 ± 1.45 years; 175.96 ± 5.24 cm; 73.16 ± 8.65 kg) were participated in a randomized placebo-control study. They consumed Pro-WPI products with 10 (T1) and 20% (T2) WPI for treatments G1 (Pro-WPI30) and G2 (Pro-WPI60), respectively, 3 times per day/5 days per week/9 weeks. The taste of Pro-WPI products was sour and cheesy, while mouthfeel was described as soft and thick because of the increased protein content in T1 and T2 (14.15 and 22.58%). The hemoglobin of the athletes increased significantly from a baseline of 12.69 g/dl to 16 and 16.66 g/dl in G1 and G2, respectively. Furthermore, the athletic performance was enhanced in vertical jump, long jump, sprinting velocity, half squats, and pushups, which reached 58.75 cm, 255 cm, 3.5 m/s, 218.75 counts, and 85 counts, respectively in G2. The healthy gut microbiome (probiotics) in parallel with increased iron bioavailability by mineral binding (whey bioactive peptides), influenced iron status and can represent a healthy practice to improve athletic anemia and performance. On the other hand, urinary albumin exceeded the border of reference range (<30 mg/g) and reached 38.25 and 44.13 mg/g in G1 and G2, while urine pH was in the normal range (4.5–8). Increased urinary albumin might be due to high rates of protein metabolism that follow high protein intake. This study provided preliminary information on metabolic responses to high protein concentrated yogurt intake in athletes who engaged in daily exercise. Further studies are needed to determine the recommended intensity of 10 and 20% Pro-WPI product consumption to achieve its benefits and avoid implications on kidney function.

## Introduction

Adequate protein is required to optimize the rate of muscle protein synthesis and achieve positive net muscle protein balance. A daily protein intake of 1.2–1.7 g protein/kg body weight (BW) has been suggested for athletes and bodybuilders to maintain muscle mass. Body proteins will be broken down to supply the energy needs of the body if dietary protein is insufficient (1). Additionally, high protein diets are increasingly popularized as a promising strategy for weight loss by providing the twin benefits of improving satiety and decreasing fat mass (2, 3). Anemia is a sign of malnutrition as red blood cells (RBCs) and Hb values are insufficient to maintain body health. Decreased Hb levels in the blood cause symptoms of fatigue, as Hb functions as an oxygen carrier (4). Athletes generally have lower hemoglobin concentrations than the general population that so-called “sports anemia”. Sports anemia is an iron deficiency caused by increased nutritional demands, dietary restrictions, decreased absorption and increased energy losses due to aerobic exercise. The anemia in athletes deserves a careful and multifactorial approach including iron bioavailability. Iron-depleted athletes improved their iron status and, possibly, physical performance, in addition to a healthy gut microbiome that also influences iron status (5).

Whey protein (WPs) are high-quality proteins that contain essential amino acids and can help in gaining muscle mass and improved performance. WP consumption has been popular, particularly among athletes (6, 7). Mineral binding properties of whey bioactive peptides and intestinal microbiota regulation increase mineral absorption in the digestive tract. Furthermore, WPs improve sensory characteristics of food, which are the result of their technological properties (8). Although dependent on the source of protein, debate on potential effects of excess protein supplement intake focused on implications on kidney function. Thus, albumin and urinary pH values are important parameters to be monitored in high-protein diet groups (2).

Probiotic microorganisms are increasingly applied to enhance the nutritional quality of cultured dairy products (9). Probiotics, such as *Lactobacilli*, obtain the ability to hydrolyze proteins present in their environment. This proteolytic activity not only generates the free amino acids needed by bacteria but also a large variety of peptides, some of which are endowed with biological activities (10). *Lactobacillus casei* is one of the most studied species in athletes and active individuals. Probiotics supplementation in athletes affects physiological changes in gut microbiota and immune function. Additionally, probiotic products contain energy and carbohydrates that are recommended to be part of overall nutrition plan of an athlete (11).

The term “functional food” is often used to refer to food products with demonstrated physiological benefits that are useful to the human body in some way (12). Functional foods designed for athletes have emerged as a novel sector of special purposes food products (13). Yogurt is considered a good vehicle for delivering innovative dairy products, in addition to the probiotic functional role. High-protein yogurts with high content of whey proteins could be beneficial in sports nutrition due to the ability of whey proteins to increase amino acids, trigger muscle protein synthesis and help in calorie-restricted diet (14, 15).

This study aimed at the production of high-protein concentrated pro-yogurt (Pro-WPI) enriched with 10 and 20% WP isolate, and investigation of the response of their daily consumption on anthropometric, hematology parameters, and athletic performance in parallel with safety consideration assessment.

## Materials and Methods

### Raw Materials

Raw milk (containing 3% fat, 3.1% protein, and 12.25% total solid) of the cow was obtained from the Faculty of Agriculture Farm, Alexandria University, Alexandria, Egypt. Skimmed milk powder (SMP) was obtained from Dairy America, Inc. (CA, United States), and it was composed of 34% protein, 51% lactose, 1.2% fat, 8.2% minerals, and 4% moisture. Whey protein isolate (WPI) was obtained from Burt Lewis Ingredients, LLC (United States), and it was composed of 91% protein, 6% moisture, and 3% fat.

Commercial freeze-dried lactic acid starter culture for direct-to-vat set (DVS) (Actimel); containing *Lactobacillus casei* CNCM-1518 (LFMP) was obtained from Danone, Egypt. In addition, yogurt commercial starter culture (Yo-Mix 495) 100 Direct Culture Unit (DCU) containing *Streptococcus thermophilus* and *Lactobacillus delbrueckii* subsp. *bulgaricus* was obtained from Danisco (Egypt). The cultures were kept at −18°C.

### Probiotic-Fermented Dairy Product WPI Enriched Preparation

The milk of cow (12.25% TS) was standardized to 14% total solids with SMP, pasteurized (82°C for 10 s, cooled to 38°C, and inoculated with Yo-Mix 495 (0.1 DCU/L w/v) for control plain yogurt preparation (16). In the case of Pro-WPI, WPI was reconstituted in the pasteurized cow milk at 4°C at ratios 10 and 20% WPI for treatments Pro-WPI30 and Pro-WPI60, respectively. The mixes were then blended using a kitchen machine at high speed for 3 min to homogenize, and aged at 4°C for 18 h. The homogenized milk was then heated at 82°C for10 s, and cooled to 38°C to be cultured with 10^9^ CFU of the probiotic culture *Lactobacillus casei* CNCM-1,518 an hour before inoculation with Yo-Mix 495 (0.1 UDC/L). After distribution in 100-g cups, the products were incubated at 38°C for 3.5 h until coagulation, and subsequently stored at 4°C.

### Chemical Composition of Pro-WPI

The chemical composition of control plain yogurt and Pro-WPI products was evaluated according to (17). pH was measured using a digital pH meter (AD3000; ADWA, Hungary).

### Microbiological Analysis

Serial dilutions of Pro-WPI yogurt samples were prepared for microbial enumeration using a buffered sodium chloride peptone solution (pH 7) containing potassium dihydrogen phosphate 3.5 g/L, disodium hydrogen phosphate anhydrous 5.8 g/L, sodium chloride 4.3 g/L, and peptone (meat) 1 g/L. Total count enumeration was performed on MRS agar (Biolife, Italy) and incubated at 38°C/72 h (18). Colony-forming units were counted and expressed as Log_10_ CFU/g.

### Sensory Evaluation

The sensory evaluation of Pro-WPI yogurt samples was assessed by a trained committee at The Faculty of Agriculture Saba Basha, Alexandria University, Egypt, on fresh products according to (19, 20), with some modifications. The 7-member sensory evaluation panel was asked to assess Pro-WPI30 and Pro-WPI60 in contrast with the control (Pro-WPI). The samples, which were stored at 4°C, were allowed to rest at room temperature (25°C) 10 min before evaluation. The samples were evaluated using a 5-point Hedonic scale (ISO, 2009). This scale consisted of the following parameters: odor (milky, yogurt, cheese, and sour milk), appearance (compact, bubbles, heterogeneous, soft, and thick), flavor (creamy, buttery, cheese, acid, sweet, bitter, and sour milk), and mouthfeel (light, thick, floury, sandy, and small lumps), accompanied by a scale of five categories as: 5 = extremely and 1= slightly.

### Experimental Procedure

#### Participant Eligibility and Baseline Assessment

Twenty-four athletes (19.6 ± 1.45 years; 175.96 ± 5.24 cm; 73.16 ± 8.65 kg) participated in this randomized double-blind placebo control study. For maintained and unified diet, lifestyle, and physical training, all the participants were accommodated at Student Housing Dormitories and studied at The Faculty of Physical Education for Men, Abu Qir, Alexandria University, Egypt. Eligibility testing (blood panel, eligibility for exercise, clinic checkup, and one-on-one interview) was finalized prior to the first exercise test. Inclusion criteria were: healthy, non-smokers, and no dietary or nutritional supplement use within 4 weeks prior to the first exercise test as described by the Austrian and German standards in sports medicine (21, 22). Standard hematology biochemical analyses were determined after overnight fast. Baseline characteristics of the participants before intervention are presented in Table 1.

**Table 1:** Chemical composition of probiotic WPI-enriched concentrated yogurt.

**Parameters**	**C**	**T1**	**T2**
Acidity	1.27 ± 0.01^a^	1.30 ± 0.02^a^	1.34 ± 0.01^a^
Protein%	3.90 ± 0.42^a^	14.15 ± 0.06^b^	22.58 ± 0.03^c^
Fat%	3.18 ± 0.01^a^	3.25 ± 0.07^a^	3.73 ± 0.09^a^
Ash%	0.51 ± 0.02^a^	1.30 ± 0.14^a^	2.15 ± 0.07^a^
TS%	14.56 ± 0.02^a^	24.80 ± 0.01^a^	34.40 ± 0.14^a^
FDM%	0.22 ± 0.01^a^	0.13 ± 0.01^a^	0.11 ± 0.01^a^
SNF%	11.40 ± 0.01^a^	21.56 ± 0.01^a^	30.67 ± 0.01^a^

*Data represented are means ± standard deviation (SD).*

*C, control plain yogurt; T1, Pro-WPI 10% WPI-enriched concentrated yogurt; T2, Pro-WPI 20% WPI-enriched concentrated yogurt.*

*FDM, fat to dry matter; SNF, solid non-fat.*

*Means in the same row followed by different letters are significantly different (p > 0.05)*.

#### Ethical Aspects

This study was performed in accordance with the World Medical Association (WMA) Declaration of Helsinki–ethical principles for medical research involving human subjects and approved by the postgraduate and research council and the ethical committee of The Faculty of Physical Education for Men, Abu Qir, Alexandria University. The athletes were briefed about the procedures of this study before signing informed consent form; all information and consent were written in the mother tongue (Arabic).

#### Study Design, Lifestyle, and Dietary Treatments

The participants were instructed to maintain their habitual diet at Student Housing Dormitories, and their lifestyle and training regimen during the 9 weeks of study. Diets were proportionally equivalent in macro-and micronutrient quantity for all the participants, containing 100% of the recommended daily allowance (RDA) for all nutrients. The athletes were strictly monitored, and compliance with the study was assessed weekly by individual interview.

The twenty-four participants were equally randomized into three groups (eight participants each). During the 9 week experimental period, the students received the products 3 times for 5 days per week in parallel with their meals. The placebo group (C) received plain control yogurt; G1 received Pro-WPI 10% WPI-enriched fermented dairy product (Pro-WPI 30), delivering 30 g WPI daily dose; and G2 received Pro-WPI 20% WP-enriched fermented dairy product (Pro-WPI 60), delivering 60 g WPI daily dose. All the participants were checked by the physician before each exercise test.

### Anthropometric Measurements

The anthropometric measurements taken for the assessment of participants were height, body weight, body mass index (BMI), basal metabolic rate (BMR), and body composition with the bioelectrical impedance analysis (BIA) method that indirectly estimates fat-free mass (FFM) and total body water (TBW) according to (23). An anthropometric rod was used for measuring the height of the participants and was recorded in cm. Measurement was taken from the floor to the vertex of the head. The participants were asked to stand erect against a wall on an even surface with feet close to each other, with the hips, back, and heels touching the wall. The body weight of the participants was measured in kg with a portable weighing machine and minimum calibration of 0.5 kg. The accuracy of the machine was checked before the subjects were asked to stand still on the platform of the machine and the bodyweight of the subjects was recorded. Body mass index (BMI), or Quetelet's index, is a measure for human body shape based on the body weight and height (kg/m^2^) of a subject. Basal metabolic rate (BMR) was considered as a dependent variable. Fat percentage, fat mass, free mass, and total body water (TBW) were considered as independent variables. To obtain accurate results, the participants were instructed to go overnight fasting and not to conduct any heavy physical activity the previous day, and their normal hydration status was ensured. Data were represented as means of n = 8 for each group ± SD.

### Laboratory Analysis

Blood samples were collected at 8.00 am on an empty stomach from each subject at the beginning and end of the experiment for complete blood count (CBC) analyses (RBCs, WBCs, Hb, platelets, MCV, and MCH) as anemia indicator. The participants were instructed to collect their urine (discarding the first urine) for determining urine color, turbidity, pH, specific gravity, glucose, bilirubin, pus cells, RBC, ketones, and albumin. All biochemical analyses were conducted using an automated chemistry analyzer (Hitachi, Tokyo, Japan) according to standard methods described in Tietz Textbook and Tietz Clinical Guide (24, 25). The data were represented as means of *n* = 8 for each group ± SD.

### Physical Performance Assessment

The participants were instructed not to perform physical training 3 days prior to any exercise test. The athletes completed a 20-min general standardized warm-up, including 15 min of general exercises (i.e., 10 min of running at a moderate pace followed by 5 min of lowerlimb active stretching) and, prior to each test, 5-min of test-specific exercises. For vertical jumping, the participants were instructed to execute a downward movement followed by complete extension of the legs with the hands fixed on the hips. The standing long jump test, also called the Broad Jump, is a common and easy to administer test of explosive leg power. An excellent result is >250 cm for men. A total of five attempts were allowed for both vertical and long jumps, interspersed with a 15-s interval. The best attempts were retained. Sprinting velocity (in 30 m) was measured in m/s, while pushups and half-squat exercises were counted. The data were represented as means of n = 8 for each group ± SD.

### Statistical Analysis

The data were presented as mean values ± standard deviation. Statistical analysis was performed by one-way analysis of variance (ANOVA) followed by Duncan's test. Differences were considered significant at (*p* < 0.05). The IBM SPSS Statistics 23 software program was used for statistical analyses [IBM Corp (2015) IBM SPSS Statistics for Windows, Version 23.0; IBM Corp, Armonk, NY, United States].

## Results and Discussion

### Characterization of Functional Pro-WPI Yogurt Products

#### Chemical Composition of Pro-WPI

The chemical composition of the Pro-WPI yogurt products compared to that of the plain control is presented in Table 1. Comparing to plain control, the significant increase in protein, fat, ash and total solids content is expected due to concentrated protein content of applied WPI (91%), fat (3%) and its minerals content (2.3%). This enrichment achieved increase in protein content of up to 14.15 and 22.58 % in T1 and T2, respectively. On the other hand, T2 contained 2-folds of WPI as T1, which led to elevated nutrient content. These results may explain the elevated SNF % (0.22, 0.13, and 0.11) and decreased FDM % (11.4, 21.56, and 30.67) for C, T1, and T2, respectively. Based on the Codex standard definition of “concentrated fermented milk,” it is hereby proposed that “high-protein yoghrt” is a yoghrt containing a minimum of 5.6% protein and <15% fat (26).

**Figure 1:**
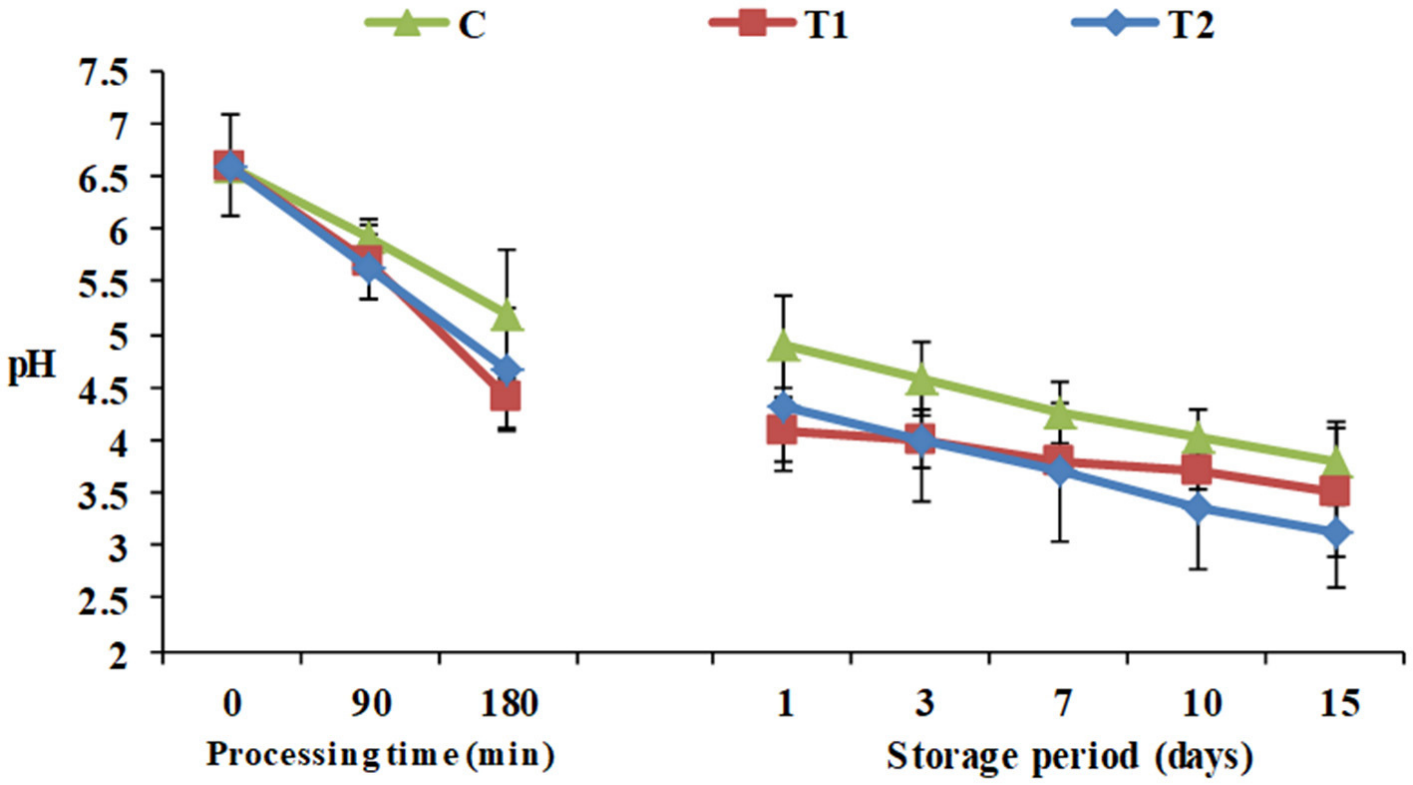
pH of yogurt products during processing and cold storage. C, control plain yogurt; T1, Pro-WPI 10% WPI-enriched concentrated yogurt; T2, Pro-WPI 20% WPI-enriched concentrated yogurt.

During processing, starting from pH 6.6 for all the yogurt products, monitoring of pH showed that the high protein Pro-WPI yogurt products showed an insignificant decrease in pH after 180 min, 4.4 and 4.68 for T1 and T2, respectively, against 5.2 for the plain control yogurt, and the same pattern was observed during the storage period, 3.5 and 3.12 for T1 and T2, respectively, against 3.78 for the control on the 15th day of cold storage (Figure 1). The results obtained were consistent with acidity values of 1.27, 1.3, and 1.34 for C, T1, and T2 respectively (Table 1). Titratable acidity was reported to increase as milk protein content increases, as milk has a base acid content attributed to proteins, minerals, and dissolved gasses, in addition to bacterial activity. On the other hand, milk protein has a strong buffering capacity that resists changes in acid or alkali content, which may have affected this increase in acidity to be insignificant (27).

#### Microbial Viable Counts

**Figure 2:**
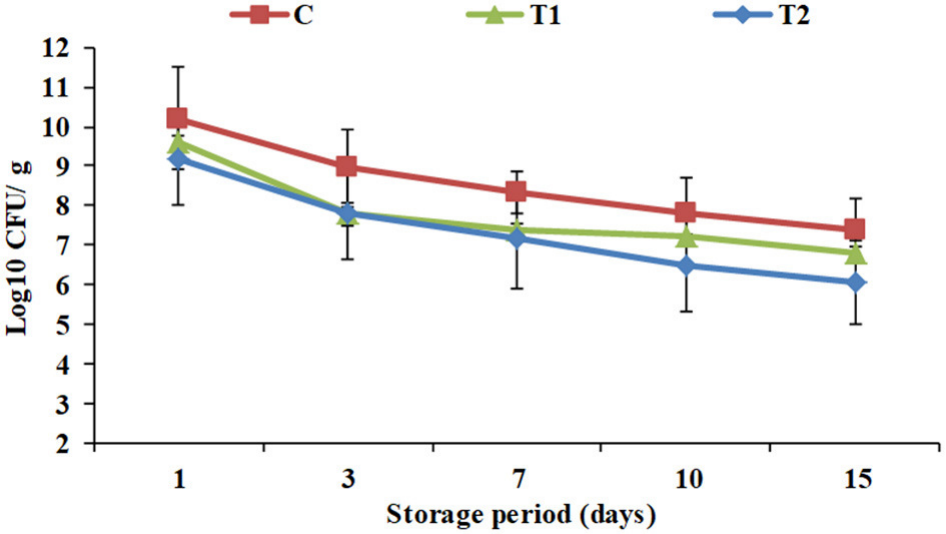
Total viable counts of yogurt products during processing and cold storage. C, control plain yogurt; T1, Pro-WPI 10% WPI-enriched concentrated yogurt; T2, Pro-WPI 20% WPI-enriched concentrated yogurt.

Total viable counts of yogurt products during processing and cold storage are exhibited in Figure 2. To receive health benefits associated with probiotics, their viability in foods is required (28). Viable counts showed a constant decline in all the products throughout the storage period. Additionally, the results showed that WPI enrichment significantly affected total viable counts in T1 and T2 (9.6 and 9.2 Log_10_ CFU/ g) compared to the control (10.2 Log_10_CFU/ g) when fresh, and along the cold storage period up to the 15th day of storage to reach 6.8 and 6.1 Log_10_ CFU/g, respectively, vs. 7.4 Log_10_ CFU/g for the control. The results were compatible with pH and acidity results (Figure 1). Similar observations were recorded by Dave and Shah (18). Nevertheless, for all the products until the 15th day of storage, viable counts were higher than 10^6^ CFU/ g, the optimum viable count to have therapeutic merit according to Dias et al. (29).

#### Sensory Parameters

**Figure 3:**
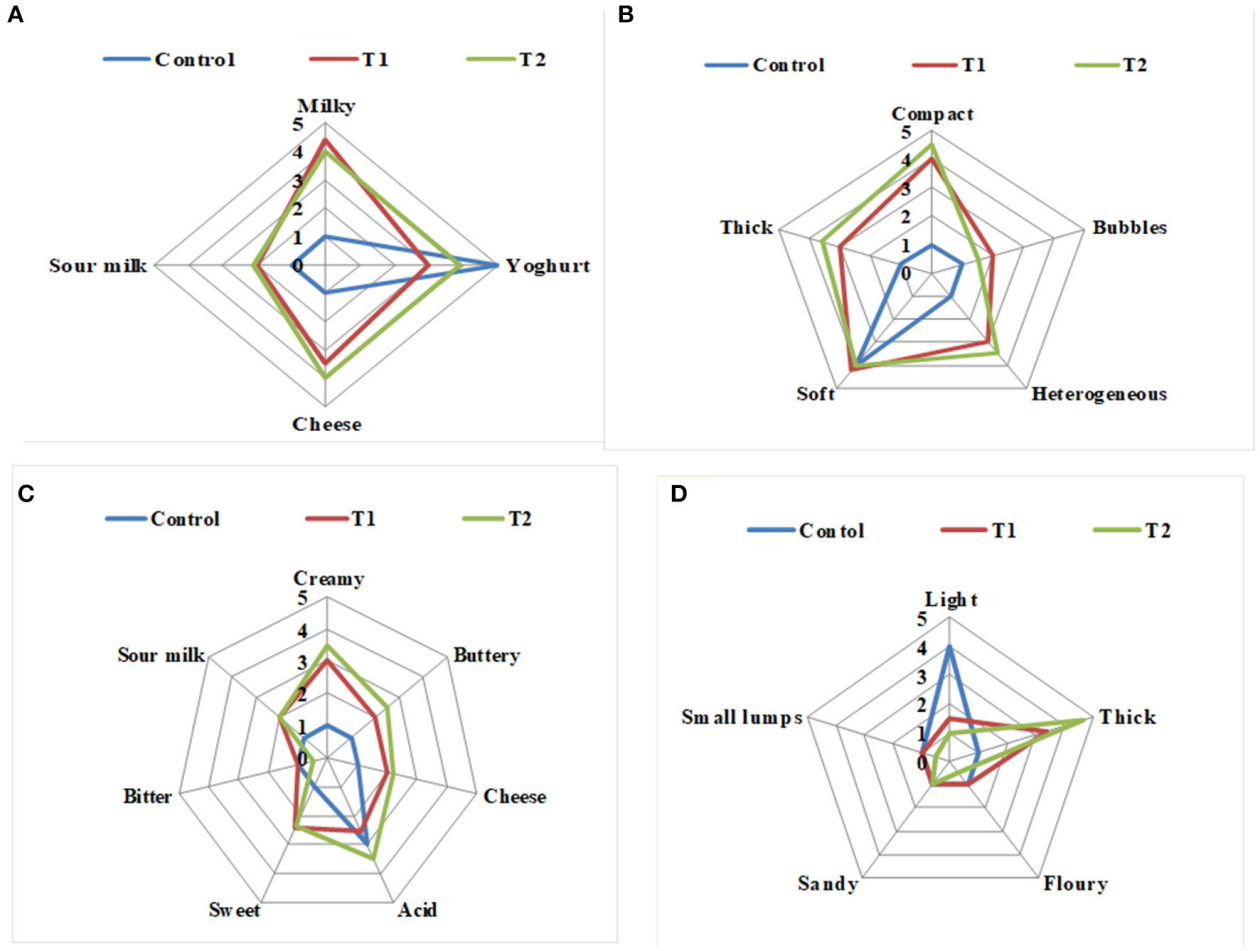
Sensory evaluation of probiotic WPI-enriched concentrated yogurt products. **(A)** Odor; **(B)** appearance; **(C)** flavor; **(D)** mouthfeel. Data represented are means of 27 processed patches (3 times per week/9 weeks). C, control plain yogurt; T1, Pro-WPI 10% WPI-enriched concentrated yogurt; T2, Pro-WPI 20% WPI-enriched concentrated yogurt.

The effects of enrichment on the sensory properties of the two Pro-WPI yogurt treatments compared to the control plain yogurt when fresh are illustrated in Figures 3A–D. The odor of the Pro-WPI yogurt T1 and T2 tended to be milky, cheesy, and sour compared to the plain control yogurt (Figure 3A). The appearance of the enriched yogurt T1 and T2 was described as thick, compact, heterogeneous, and soft compared to the plain control yogurt that was soft (Figure 3B). Concerning flavor; the Pro-WPI products T1 and T2 tended to be creamy, buttery, cheesy, and sweet with acid flavor, while the acidic flavor of the plain control yogurt was the most significant (Figure 3C). The mouthfeel of the Pro-WPI yogurt products was thick compared to the light mouthfeel of the plain control yogurt (Figure 3D). The results showed a reflection of the chemical composition on the organoleptic properties of the enriched Pro-WPI products (Table 1). The obtained results came inconsistency with (14), who also stated that attributes, such as thickness, creaminess, and softness, are important drivers of liking high-protein yogurts.

### Baseline Characteristics and Hematology Biochemical Parameters

Baseline characteristics, anthropometric measurements, and hematology biochemical parameters of the 24 athlete-participants before the intervention are summed in (Table 2). Due to unified conditions, low deviations were recorded in terms of age and BMI of the athlete-participants with mean values of 19.6 years and 23.54 kg/m^2^, respectively. The results showed that the mean hemoglobin level (12.69 g/dl) was less than the reference range (14–18 g/dl), and that the red blood cell (s) counts were on edge of the least reference range. The other hematology biochemical parameters were within the average range. The World Health Organization defines anemia as blood hemoglobin values of <13 g/dl (30).

**Table 2:** Baseline characteristics, anthropometric measurements, and placebo biochemical parameters.

**Variable**	**Unit/adult reference range**	**Results**
Age	Year	19.6 ± 1.45
Height	cm	175.96 ± 5.24
Weight	kg	73.16 ± 8.65
BMI	kg/m^2^	23.54 ± 2.12
BMR	J/(h·kg)	1,890.48 ± 174.48
Fat %	%	13.13 ± 4.67
Fat mass	kg	9.88 ± 4.39
FFM	Kg	63.27 ± 5.67
TBW	L	46.59 ± 4.30
RBC	4–5 X 10^6^ /μL	3.94 ± 0.53
Hb	14–18 g/dl	12.69 ± 1.01
WBC	4.5–11.0 × 10^3^ /μL	7.32 ± 1.74
Mono	2–10%	6.09 ± 0.79
Lymph	20–40%	38.26 ± 4.54
Plt	150–450 × 10^3^ /μL	198.69 ± 28.93
MCV	80–100 fl	87.82 ± 5.66
MCH	28–34 pg	28.57 ± 0.79

*Data represented are means of (n = 24) ± SD. Reference range for clinical chemistry parameters (26). BMI, body mass index; BMR, basal metabolic rate; FFM, fat-free mass; TBW, total body water*.

### Effect of Pro-WPI Functional Products on the Athlete-Participants

#### Effect on Anthropometric Measurements and Hematology Parameters

The effect of PRO-WPI products on anthropometric measurements and hematology biochemical parameters are shown in (Table 3). BMR reflected that its value of G1 (1,854.25 Kcal/day) was significantly less than that of the placebo group (1,892.14 Kcal/day), and that the value of G2 was highest (2,040 Kcal/day). These results were reflected on weight and BMI of G1 (10% WPI) that were significantly less compared to those of the placebo group that ate plain yogurt. High-protein diets are increasingly popularized as a promising strategy for weight loss by providing the twin benefits of improving satiety and altering metabolism, leading to decreased fat mass (2, 3). Increased BMR due to increased fat content of Pro-WPI60 (20% WPI) (Table 1) may be a reason for significant elevation in weight, fat, fat mass, fat-free mass (FFM), and, consequently, BMI (79.64 kg, 13.09%, 10.42 kg, 69.23 kg, and 25.1 kg/m^2^, respectively) in G2 members compared to G1 (69.19 kg, 8.35%, 5.87 kg, 63.57 kg, and 22.59 kg/m^2^).

**Table 3:** Effect of Pro-WPI products on anthropometric measurements and hematology biochemical parameters.

**Variable**	**Unit/adult reference range**	**Placebo (C)**	**Group 1**	**Group 2**
Height	cm	175.43 ± 5.62^b^	174.8 ± 4.52^b^	177.62 ± 5.88^a^
Weight	kg	74.33 ± 10.92^b^	69.19 ± 3.21^c^	79.64 ± 5.47^a^
BMI	kg/m^2^	24.19 ± 2.57^a^	22.59 ± 1.42^b^	25.10 ± 1.13^a^
BMR	(Kcal/day)	1,892.14 ± 227.34^b^	1,854.25 ± 84.17^b^	2,040 ± 146.60^a^
Fat	%	14.91 ± 4.50^a^	8.35 ± 3.59^b^	13.09 ± 3.28^a^
Fat mass	kg	11.40 ± 4.98^a^	5.87 ± 2.79^b^	10.42 ± 2.70^a^
FFM	kg	62.93 ± 7.24^b^	63.572.29^b^	69.23 ± 5.27^a^
TBW	L	45.96 ± 5.25^b^	45.58 ± 2.24^b^	49.55 ± 3.99^a^
RBC	4–5 × 10^6^ /μL	4.91 ± 1.00^a^	5.44 ± 0.67^a^	5.89 ± 0.57^a^
Hb	14–18 g/dl	14.86 ± 1.62^b^	16.01 ± 2.57^a^	16.66 ± 2.18^a^
WBC	4.5–11.0 × 10^3^ /μL	7.65 ± 1.55^b^	8.62 ± 1.21^a^	8.70 ± 1.08^a^
Mono	2–10%	6.86 ± 0.90^b^	7.63 ± 1.19^a^	7.63 ± 0.74^a^
Lymph	20–40%	37.57 ± 5.06^b^	40.63 ± 0.74^a^	41.75 ± 1.16^a^
Plt	150–450 × 10^3^ /μL	214.28 ± 34.08^c^	250.62 ± 41.44^a^	243.62 ± 34.14^b^
MCV	80–100 fl	87.29 ± 6.70^c^	90.13 ± 6.33^b^	92.63 ± 7.07^a^
MCH	28–34 pg	29.21 ± 1.07^a^	29.55 ± 1.04^a^	30.38 ± 2.26^a^

*Data represented are means of n = 8 for each group ± SD. Placebo group (C), received control plain yogurt; G1, received Pro-WPI 10% WPI-enriched concentrated yogurt (30 g WPI daily dose); G2, received Pro-WPI 20% WPI-enriched concentrated yogurt (60 g WPI daily dose). BMI, body mass index; BMR, basal metabolic rate; FFM, fat-free mass; TBW, total body water. Means in the same row followed by different letters are significantly different (p > 0.05)*.

Concerning hematology parameters; compared to Hb baseline (12.69 g/dl) (Table 2), all the fermented products (Pro-WPI, Pro-WPI 30, and Pro-WPI 60) in placebo, G1, and G2 achieved a significant increase in Hb to 14.86, 16, and 16.66 g/dl, respectively. Similarly, in RBC, a baseline was 3.94 × 10^6^/μl, which was elevated to 4.91, 5.44, and 5.89 × 10^6^/μl, respectively. Fermented milk contains lactic acid and other organic acids that are produced during fermentation, and it increases the absorption of iron especially when consumed at mealtimes (31). *Lactobacilli* sp. are featured with developed ability to hydrolyze proteins in their environment. This proteolytic activity not only generates free amino acids needed by bacteria but also a large variety of peptides, some of which are endowed with biological activities. The so-called “bioactive peptides” (BAPs) are interesting from nutrition and healthcare perspectives (10). Probiotic bacteria were reported to enhance iron absorption and utilization because of bioactive factors of fermented milk; hence they enhance and protect from iron-deficiency anemia (32–34). On the other hand, whey protein-derived bioactive peptides were reported to show considerable capacity for binding properties for cations, such as calcium, iron, and zinc (35), and the potential to regulate the intestinal microbiota that encourages mineral absorption by the digestive tract (8), which can explain the significant increase of Hb of G1 and G2 who ate the PRO-WPI products compared to placebo. The obtained results reflected that healthy gut microbiome (probiotics), in parallel with increased iron bioavailability by mineral binding (whey bioactive peptides), influences iron status and can represent a healthy practice to improve athletic anemia (5). Slight increases observed in WBC and lymph were previously reported because of immune modulation by starter culture and probiotic bacteria (36). Significant increases in platelets up to (250.62 and 243.62 × 10^3^ /μl) in G1 and G2, respectively, comparing to placebo (214.28 × 10^3^ /μl) came in accordance with Mazzuca et al. (37), who referred these results to the whey proteins ability to improve vitamin B12 and folate absorption that consequently could increase platelet count and reduce platelet depletion as well. No significant differences were obtained with respect to MCV and MCH.

#### Effect on Athletic Performance

Influences of the Pro-WPI products on athletic performance are exhibited in Table 4. Significant enhancements were recorded for most of the tested athletic performance parameters; vertical and long jumps, half squat, and pushup counts. Whey proteins were reported to increase protein synthesis and recovery of athletes after training and competition (8). Moreover, probiotic supplementation for athletes with the key term “probiotic athlete” was most commonly studied applying species such as; *Lactobacillus casei, L. fermentum, L. acidophilus*, and *L. rhamnosus*, to determine the effects of these probiotics on clinical measures and immune function in a placebo controlled design experiments (11). As the Pro-WPI products contain both whey proteins and probiotics, they can have a positive effect on athletic performance and can be recommended.

**Table 4:** Influences of Pro-WPI products on athletic performance.

**Parameters**	**Unit**	**Placebo (C)**	**Group 1**	**Group 2**
Vertical jump	cm	53.29 ± 4.46^b^	59.75 ± 5.44^a^	58.75 ± 4.46^a^
Long jump	cm	234.57 ± 9.97^b^	256.25 ± 15.06^a^	255.00 ± 17.11^a^
Sprinting velocity (30 m)	m/s	3.63 ± 0.30^a^	3.51 ± 0.25^b^	3.50 ± 0.35^b^
Half squat	count	163.43 ± 27.34^c^	210.38 ± 26.85^b^	218.75 ± 29.00^a^
Pushups	count	65.00 ± 13.23^b^	86.88 ± 6.68^a^	85.00 ± 15.81^a^

*Data represented are means of n = 8 for each group ± SD. Placebo group (C), received control plain yogurt; G1, received Pro-WPI 10% WPI-enriched concentrated yogurt (30 g WPI daily dose); G2, received Pro-WPI 20% WPI-enriched concentrated yogurt (60 g WPI daily dose). Means in the same row followed by different letters are significantly different (p > 0.05)*.

### Safety Considerations of Pro-WPI Product Consumption

Urine biochemical assessment of the participants after consumption of the PRO-WPI products is exhibited in Table 5. Postures or exercises were reported to cause elevation, so experts recommend first-morning spot collection for screening purposes (38). Nevertheless, protein-rich diets are acidogenic because of the release of excessive non-carbonic acids, which are produced by the metabolism of protein (1), but pH and other examined urine parameters showed to be comparable to the placebo group except for significant differences recorded for albumin concentrations. The consumption of high amounts of proteins was reported to contribute to changes in urinary composition in high-protein dieted rats which vary according to the protein type either; dairy, non-dairy, animal, or vegetable protein (6). Microalbuminuria (MA) refers to increase in urine albumin excretion, and consumption of dietary protein is one of its reported causative factors (39). In the 1980s, McCarthy reported that daily protein intake of up to 2 g/kg was not detrimental to kidney function in male athletes (7, 40). As the daily intake of PRO-WPI products for the athletes participants (0.82 g/kg), caused elevated concentration of albumin (38.25 and 44.13 mg/g in G1 and G2, respectively) compared to placebo group (8.57 mg/g), exceeding the reference range (<30 mg/g), it is recommended to reduce the daily intake of PRO-WPI (10%) to be twice a day on daily basis. However, restriction of dietary protein intake (DPI) reduces urinary albumin levels (39).

**Table 5:** Urine biochemical assessment of the participants.

**Variable**	**Unit/adult reference range**	**Placebo (C)**	**Group 1**	**Group 2**
Color	Yellow	1.00 ± 0.00^a^	1.00 ± 0.00^a^	1.00 ± 0.00^a^
Turbidity	Clear/Cloudy	2.00 ± 0.00^a^	2.00 ± 0.00^a^	2.00 ± 0.00^a^
pH	4.5–8	6.10 ± 0.48^a^	5.91 ± 0.46^a^	5.88 ± 0.35^a^
Specific gravity	1.005–1.030 (g/mL)	1.142 ± 0.078^a^	1.162 ± 0.050^a^	1.162 ± 0.051^a^
Glucose	Negative (mg/dL)	Negative	Negative	Negative
Bilirubin	0.3– 1.2 (mg/dL)	Negative	Negative	Negative
Pus cells	0–5/hpf	6.71 ± 0.42^a^	4.25 ± 0.70^b^	4.00 ± 0.00^a^
RBC	Negative	1.14 ± 0.37^a^	1.00 ± 0.00^a^	1.00 ± 0.38^a^
Ketones	Negative (mg/dL)	Negative	Negative	Negative
Albumin	<30 (mg/g)	8.57 ± 1.69^c^	38.25 ± 4.33^b^	44.13 ± 2.94^a^

*Data represented are means of n = 8 for each group ± SD. Placebo group (C), received control plain yogurt; G1, received Pro-WPI 10% WPI-enriched concentrated yogurt (30 g WPI daily dose); G2, received Pro-WPI 20% WPI-enriched concentrated yogurt (60 g WPI daily dose). Means in the same row followed by different letters are significantly different (P > 0.05). Color, yellow (1: light/pale to 1.5: dark/deep amber); turbidity (1.5–2: clear to 1–1.5: Cloudy); hpf, high-power field*.

## Conclusion

High-protein concentrated pro-yoghrt (Pro-WPI) enriched with 10 and 20% WPI showed acidic flavor with soft thick texture because of increased protein content. When daily consumed by the 24 athletes, the products enhanced the hemoglobin significantly and consequently improved both athletic anemia and performance because of both whey proteins and probiotics. Urinary albumin exceeded the border of the reference range because of high rates of protein intake, while urine pH was normal. This study provided preliminary information on high-protein concentrated yogurt intake in athletes who engaged in daily exercise. Further studies are needed to determine the recommended intensity for Pro-WPI products consumption to achieve their benefits and avoid implications on kidney function.

## Data Availability Statement

The raw data supporting the conclusions of this article will be made available by the authors, without undue reservation.

## Ethics Statement

The studies involving human participants were reviewed and approved by Institutional Committee of Faculty of Physical Education, Alexandria University, Egypt. The patients/participants provided their written informed consent to participate in this study.

## Author Contributions

MG, A-EE, and AD were responsible for conceptualization, experimental design, methodology, and data curation and analysis. MG, MA, and AH performed formal analyses. MG, AH, and AD wrote the original draft of the manuscript. All authors revised, edited, and confirmed the manuscript.

## Conflict of Interest

The authors declare that the research was conducted in the absence of any commercial or financial relationships that could be construed as a potential conflict of interest.

## Publisher's Note

All claims expressed in this article are solely those of the authors and do not necessarily represent those of their affiliated organizations, or those of the publisher, the editors and the reviewers. Any product that may be evaluated in this article, or claim that may be made by its manufacturer, is not guaranteed or endorsed by the publisher.
